# Qualitative study of views and experiences of performance management for healthcare-associated infections

**DOI:** 10.1016/j.jhin.2016.01.021

**Published:** 2016-09

**Authors:** L. Brewster, C. Tarrant, M. Dixon-Woods

**Affiliations:** Department of Health Sciences, University of Leicester, Leicester, UK

**Keywords:** Performance management, Infection prevention and control, Hospital management, Qualitative

## Abstract

**Background:**

Centrally led performance management regimes using standard setting, monitoring, and incentives have become a prominent feature of infection prevention and control (IPC) in health systems.

**Aim:**

To characterize views and experiences of regulation and performance management relating to IPC in English hospitals.

**Methods:**

Two qualitative datasets containing 139 interviews with healthcare workers and managers were analysed. Data directly relevant to performance management and IPC were extracted. Data analysis was based on the constant comparative method.

**Findings:**

Participants reported that performance management regimes had mobilized action around specific infections. The benefits of establishing organizational structures of accountability were seen in empirical evidence of decreasing infection rates. Performance management was not, however, experienced as wholly benign, and setting targets in one area was seen to involve risks of ‘tunnel vision’ and the marginalization of other potentially important issues. Financial sanctions were viewed especially negatively; performance management was associated with risks of creating a culture of fearfulness, suppressing learning and disrupting inter-professional relationships.

**Conclusion:**

Centrally led performance management may have some important roles in IPC, but identifying where it is appropriate and determining its limits is critical. Persisting with harsh regimes may affect relationships and increase resistance to continued improvement efforts, but leaving all improvement to local teams may also be a flawed strategy.

## Introduction

As in other countries worldwide, the landscape of infection prevention and control (IPC) in the English National Health Service (NHS) has been shaped in recent years by extensive policy-driven performance management. Much of this recent history in England can be dated to 2000, when a highly critical National Audit Office report identified poor surveillance that relied primarily on voluntary reporting and noted that healthcare-associated infections (HCAIs) had become seen as a problem to be regretted but tolerated.^1^ The policy interventions that followed initially involved relatively gentle moves that encouraged organizations to recognize the need for change and for appropriate actions in order to improve.^2^ However, by the mid-2000s public concern about HCAIs had grown, fuelled by media reporting, patient pressure groups, litigation and complaints, and reputational damage associated with specific cases of failings.^3^, ^4^, ^5^ In response, the Department of Health seized the initiative for making change happen. From this point onwards, performance management, led from the centre, became a prominent feature of IPC in England (Table I).

The characteristics of this performance management are similar to those used in other public services, including establishment of performance standards and measurement as well as the introduction of incentives and sanctions.^6^ Healthcare providers in England are now subject to multiple forms of oversight and control. Surveillance of infection rates, standard setting through the National Institute for Health and Care Excellence (NICE), public reporting, and target-driven approaches (for example, based on key performance indicators for specific infections) accompanied by financial and reputational penalties have combined with a much more forceful legal and regulatory framework.^7^, ^8^, ^9^

With the Health Act 2006, prevention and control of HCAI became statutory duties for NHS trusts. These duties were further strengthened by the Health and Social Care Act 2008, which made it a legal requirement of providers' registration with the Care Quality Commission (CQC) that they comply with a code of practice for HCAI.^9^ Providing for a range of punitive sanctions, the code is in marked contrast to previous guidance, which tended to have a somewhat voluntary character. It specifies ten duties with which registered providers are expected to comply, including the establishment of systems to manage and monitor the prevention and control of infection, to provide a clean and appropriate environment, and to ensure that all staff are suitably educated. The CQC may inspect or otherwise audit providers, and is able to impose a wide range of sanctions in the event of non-compliance, up to and including de-registration.

At the same time, other agencies and bodies have become more actively engaged in IPC issues, especially since the 2012 Health and Social Care Act. These include (but are not limited to) NHS England and its Clinical Commissioning Groups (CCGs), who can enforce IPC measures through contracts and for whom incidence of *Clostridium difficile* and meticillin-resistant *Staphylococcus aureus* (MRSA) is part of the CCG outcomes indicator set reported to NHS England.^10^, ^11^

Substantial reductions in HCAI rates in England have occurred over the last 15 years.^12^ However, performance management of IPC has been the focus of considerable concern, not least because the broader literature has identified many of the unintended consequences that tend to be associated with ‘targets and terror’ regimes.^13^, ^14^, ^15^, ^16^ Yet the perspectives of healthcare workers who are subject to these regimes have remained largely neglected: the benefits and disadvantages of performance management for HCAI have instead been ‘read off’ surveillance data and other sources. Using qualitative methods, we sought to characterize the views and experiences of regulation and performance management relating to IPC of healthcare workers and managers in English hospitals.

## Methods

Two data sources (Source 1 and Source 2) were used. Source 1 involved case studies of two English NHS hospitals that were chosen to reflect different organizational structures, patient numbers, and *C. difficile*, MRSA, and mortality rates. Semi-structured interviews were conducted with a purposive sample of staff, including frontline staff sampled from eight wards across the organizations, and key executive-level staff. All interviews were audio-recorded, transcribed verbatim, and anonymized. A list of themes generated from a literature review was used as an initial coding frame. Further themes were generated inductively, using open coding and organization of open codes into higher-order categories.^17^

Source 2 was a study of efforts to prevent central line infections in 19 intensive care units (ICU) in England, involving interviews with a purposive sample of staff within each participating ICU.^18^ Data directly relevant to performance management and IPC were extracted and used to build and extend the themes identified from Source 1. Data from both sources were then synthesized to form a single analysis.

Source 1 was designated as service evaluation under the NHS Research Governance Framework; ethical approval for Source 2 was gained from Berkshire NHS Research Ethics Committee.^19^

## Results

Source 1 included interviews with 41 staff members: 37 frontline staff including nurse consultants, matrons, ward managers, nurses at all levels, healthcare assistants, and infection control specialists, and four executive-level staff. Source 2 included interviews with 98 individuals: the majority were clinical staff, including consultant and registrar-level physicians, service improvement leads, specialist nurses, infection control practitioners, and managers. Across both sources we identified themes relating to both positive and negative consequences of performance management.

### Positive impacts of performance management

Participants (especially but not only in Source 1) identified several positive consequences of performance management, including raising the priority of IPC, driving the establishment of formal structures and lines of accountability, making IPC a collective responsibility, and generating data to drive improvement.

#### Raising the priority given to IPC

External pressure to improve as a result of centrally driven performance management had been felt throughout all organizations, and was identified by participants as having stimulated action that had improved patient care. Some participants argued that changes would not have occurred without the external pressure, since there had previously been insufficient internal imperative within organizations to prevent and control infection appropriately. The use of financial and reputational sanctions was identified by participants as especially important in attracting the attention of senior management and increasing the priority afforded to IPC.

One of the great successes obviously in infection control was the reduction targets, they were very top-down and you will do this. And if you don't we'll potentially fine you and we'll name and shame you and the chief executive who's responsible. And they worked. (Infection control nurse, Source 1)

Certainly five or ten years ago people weren't so interested in infection control, unless it related to a specific problem in their department, [like] dealing with an outbreak … We've managed to, over the years, introduce things that people would have thought maybe unthinkable a few years ago … So I think there is a greater acceptance now of the importance of infection control than there was. (Consultant microbiologist, Source 2)

#### Formal structures and lines of accountability

A further positive impact of the performance management regime was seen by some participants to lie in the establishment of formal leadership and governance structures for IPC, stimulated by legal and other requirements, so that lines of accountability and resourcing were now much more explicit and clear. Many different forms of accountability had been established, including but not limited to audits of practices, feedback of infection data on a ward-by-ward basis, and root-cause investigations into specific cases.

Within this trust, if we have any bacteraemia – if it's MRSA, then that goes to the chief exec. But you're expected to be accountable for what happens to your own bacteraemia as well. (Consultant, Source 2)

If we do have some sort of blip, we have C. diff summits which we're all called to and challenged on, and [we] would look at all our cleaning records and so on. (Matron, Source 1)

Executive-level participants also reported that external reputational pressures, including media attention, had helped to propel IPC on to the agendas of organizational board meetings and to ensure that IPC interventions were explicitly resourced.

The pressure [to improve IPC performance] is essentially because of the […] reputation of the ward, of the department, of the hospital. (Executive team member, Source 1)

#### IPC as a collective responsibility

One of the especially positive effects of performance management of HCAI reported by participants was a new sense of acceptance of the importance of IPC and the need for it to be a collective responsibility: IPC had become increasingly understood as requiring everyone's co-operation and support.

Ownership [of IPC tasks] for more people, and the understanding that it matters, what they're doing. It's got to be everyone's problem. (Matron, Source 1)

Over the past seven to eight years, [we have] made them realise that infection control is not the infection control team's responsibility alone. It is everyone's responsibility. Take ownership, be accountable, and act on failures. And we help and support and do the surveillance constantly, that's our role. (Consultant, Source 2)

#### Data to drive improvement

Surveillance of infection, stimulated by both national data collection programmes and local audits, was reported by participants to have increased in scale to the point where it was a normalized aspect of routine work. Again, positive consequences included new recognition of HCAI problems that had previously remained obscured, identification of where organizations were performing poorly in comparison with peer organizations, and the ability to monitor improvement.

About four years ago or so they had a big problem in MRSA bacteraemias, I think it was it was quite bad compared to some of the other [hospitals]. (Infection control nurse, Source 2)

Surveillance data were also seen by participants as having value in motivating frontline improvement. Multiple methods of making data available were described including charts, dashboards, and public displays. Data were used in positive ways to encourage and reward staff and to instil a sense of professional pride.

The cleaner that I spoke to […] said, ‘before I started their statistics weren't very good, now we're always 99, 100% and never drop below that’. And she took me out and showed the certificate she'd put out by the families' waiting room which acknowledged her for good performance. (Observations, Source 2)

We developed and introduced an infection control accreditation programme, and to be accredited, the ward has to work through a series of standards. Their accreditation is judged by an infection control nurse ultimately, and they're really proud, and that's driven really good practice. (Executive team member, Source 1)

### Negative impacts of performance management

Though externally driven performance management was recognized to have generated pressures to reduce HCAIs, it was not experienced by participants as wholly benign. It was reported to have produced a range of unwanted consequences including tunnel vision, a culture of fearfulness, and the introduction of conflict and tension into working relationships.

#### Tunnel vision

Participants suggested that risks arose when performance management regimes targeted only specific infections (such as MRSA), or specific infection routes – resulting in neglect of other types of infections or of issues that staff saw as equally or more important.

I think there are a lot worse things [than central line infections] – wound-related infections for example – that emphasis should be given to. (Consultant, Source 2)

We try and continue to improve performance on key organisms but also [need] to be looking at a wider range of issues which perhaps haven't been tackled quite so consistently as the key headline organisms. That would be around things like surgical site surveillance, urinary tract infections, hospital-acquired pneumonia/chest infections. (Executive team member, Source 1)

Participants also expressed a concern that the drive to produce data towards managerially set goals could easily displace concerns about the patient.

I think the unfortunate thing with all of these issues often is that we forget about why we're doing certain things … you get the feeling it's actually not to do any more with the patients, it's got to do with your tick boxes, your numbers. (Consultant, Source 2)

If we've lost sight of the fact that we're here for patients, then that's a bit of a problem, isn't it? (IPC nurse, Source 1)

#### Culture of fearfulness

The risk of sanctions for poor performance meant that a major consequence of performance management was a culture of fearfulness around IPC. In line with the national approach of top-down, mandated imperatives from the centre, organizations often increased the managerial attention given to IPC, essentially by reproducing top-down approaches of their own. Participants described punitive managerial behaviours, which they saw as being driven by externally imposed targets and the reputational consequences of public reporting. Words such as ‘slap’, ‘whip’, ‘telling off’ and ‘disciplinary’ recurred in the interviews:

So if I get one C. diff in ITU the [organization] board is down on me … yeah, they come down very heavily on me, say ‘why did it happen, how did you let this happen?’ (Microbiologist, Source 2)

If we're not doing as well as we should be doing […] then we start getting our wrists slapped. (Senior nurse, Source 2)

The effects of this culture of increased fearfulness were multiple. In some cases, it led to generalized anxiety about data and the purposes for which it could be appropriated. Staff were concerned about the linking of performance data with personal or financial sanctions, which they saw as impeding improvement efforts and suppressing a learning culture. Staff recognized that punitive performance management approaches could taint subsequent quality improvement efforts even when these were based on voluntary, collaborative principles.

This [organization] will do a root cause analysis if there is a bacteraemia and […] they do feel like a witch-hunt. The person who put in that cannula or whatever will be given a good slap verbally, and it is not the caring, sharing, let's improve behaviour. (Consultant, Source 2)

It's all target driven, and if you don't achieve your targets you get penalised financially. It seems you're not doing well, so we'll take more money off you so you can do even worse. It's not like: well what do you need and we'll give you some more money and then we expect you to improve. We get penalised for it. (Senior nurse, Source 1)

If [staff] perception is that this data will be seized upon and used against them it makes people much more reluctant to engage in the whole process. (Consultant, Source 2)

#### Conflict and tension

Despite the increasing acceptance of IPC as a collective responsibility, some participants suggested that performance-led initiatives tended to reduce rather than enhance co-operation between teams and professional groups by transforming IPC into an area of conflict and tension. IPC teams that were seen to be heavy-handed or confrontational were particularly resented, and there was a risk that IPC practitioners could come to be seen by other staff as a threatening presence in their wards or units.

Infection control initiatives usually boil down to a ‘you are terrible, do something about it’ approach. You get a horrible e-mail, saying like ‘you are crap again’. (Consultant, Source 2)

Some [IPC nurses] unfortunately get labelled as rottweilers because they come on and are very demanding – you have not done this, you will do that. I think the staff find it very difficult […] so the relationship between Infection Control and staff in general is a bit strained at times. (Senior nurse, Source 1)

My perception is that people panic when they see infection control come, and that they're being spied on. (Nurse, Source 1)

Tensions were also evident between the medical and nursing professions more generally. Nurses often considered that it was their responsibility to ensure that IPC requirements were met, and they were answerable for audits and for ensuring compliance. But some described feeling powerless to influence the behaviour of their medical colleagues, and felt resentful of the enforcer role that they felt obliged to assume.

The doctors are not the best at following the infection control procedures when you've got the barrier rooms. They'll walk out with their gloves and their apron on and you're like ‘oh my goodness don't do that!’ But what can you say? (Clinical support worker, Source 1)

Why should it be up to the nurses to do it? Why can't the doctors be trained so they can do it adequately? If I had to do an aseptic technique and I needed somebody at the end of the bed to tell me what to do, I shouldn't be doing it. (Nurse, Source 2)

Doctors, on the other hand, were often aggrieved at the perceived erosion of their professional autonomy. Some reported feeling humiliated and resentful at how they were treated as miscreants and at what they saw as an assault on their professional integrity and dignity. A particular source of grievance centred on how the rise of managerial power had, as they saw it, rendered them subservient to others who lacked the appropriate knowledge and expertise.

You know we've got a lot of management saying what we should do and shouldn't do now and there is definitely a move to, you know, take away professionalism altogether ... And I think if you want somebody to be professional you teach them and you bring them up to take responsibility for their actions. (Consultant, Source 2)

## Discussion

Our analysis suggests that externally led performance management was broadly recognized by clinical staff and managers in English acute hospitals as having raised the profile of IPC and supported the establishment of organizational accountability structures, with positive consequences relating to clarity of goals and responsibilities, use of data, and resourcing. Performance management approaches were not, however, seen as wholly benign by participants. Significant negative consequences were described, including tunnel vision and the engendering of a culture of fear and of conflict between professional groups. Thus, though centrally driven performance management approaches may have achieved much, these findings suggest that more effort should be focused on when and how it can most appropriately be applied.

The strength of this study lies in the synthesis of qualitative data across two large datasets, including interviews with staff involved in IPC at all levels, and its ability to combine data from a specific clinical setting (intensive care in 19 organizations) with a broader perspective across two organizations that were studied in-depth. A limitation of the study is that our focus on IPC did not include antimicrobial resistance, which has a specific history of management and regulation. The study might have benefited from including more managers and clinical perspectives from a wider range of specialties; our sample construction in Source 2 was biased towards ICUs, where participants may be especially focused on a narrow range of specific types of infections (e.g. intravenous-related).

In clarifying the role of performance management for IPC, it is worth being clear about the features that appear to be implicated in its successes and how they might be preserved while minimizing the more adverse consequences. Chief among the beneficial aspects of performance management appears to be its ability to draw attention to problematic areas of practice and to mobilize action.^20^ Participants in our study were clear that external mandates had created a stimulus for improvement. It appeared that the introduction of performance management had produced agenda-setting effects that converted HCAI from a problem that had become rather neglected and under-resourced into a social problem demanding a solution in the face of intense public and political pressure.^20^, ^21^ National standards reinforced by the establishment and maintenance of systems for monitoring had a key role in making local problems visible and hence actionable.^22^

The positive effects of performance management described by participants in our study are readily observable from the empirical evidence. For instance, the introduction of mandated surveillance for *C. difficile* allowed the scale of the problem and any improvement over time to be assessed. The data showed a peak of >55,600 cases in patients aged >65 years in 2006; by 2010, numbers had decreased by 54% to >25,500.^12^ Similarly, reported MRSA bacteraemias showed an 87% fall from >7000 in 2001/2 to 924 in 2012/13.^23^ However, further gains from surveillance and benchmarking are likely to require attention to the formidable technical and resource challenges (especially for some kinds of HCAIs), to the need for data to be locally credible, and to issues of fairness and comparability across different institutions.^18^, ^24^

A second important feature described by participants is clarity about organizational accountability for goal achievement. Hospitals are complex organizations characterized by what is described in the regulation and governance literature as ‘the problem of many hands’, which often makes it difficult to determine who is responsible for what.^25^ Reform of systems of governance has now made hospital management teams responsible for addressing issues of infection control.^7^ As described by participants, the increased managerial attention given to IPC has had many positive consequences. But unless carefully selected and optimized, managerially led goal-directed behaviour may produce the tunnel vision effect described by participants, known in the economics literature as ‘effort substitution’.^16^ Here, attention becomes narrowly focused on the thing being measured, to the exclusion of other equally (or perhaps even more) valuable issues. Setting a target in one area may therefore mean that untargeted areas are neglected: the use of MRSA as a performance target forced hospitals to prioritize an infection that accounts for only 2% of HCAI, and was implicated in outbreaks of *C. difficile* at Stoke Mandeville hospital between 2003 and 2005 when this infection was not subject to performance management.^26^, ^27^ Thus, while MRSA bacteraemias and other specific infections remain an important area for attention, their continued status as a measurable and reportable infection creates risks that those pathogens that are unmonitored – such as norovirus – rise in rate and significance, or that other deserving areas for intervention (such as antibiotic stewardship) are crowded out.

Other features of performance management are potentially even more contentious. Public reporting was understood by our study participants to have yielded some positive impacts, though they were not uniformly good. Financial sanctions, on the other hand, were much resented by participants. Consistent with their views, there is little published evidence to justify financial penalties. Two recent US studies examining a non-payment policy found that it had no measurable effect on central line infections or other HCAI rates.^28^, ^29^ Further, financial penalties appear to increase the risk of gaming; some have argued that infection rates may say more about willingness to report than underlying rates of harm.^30^, ^31^ Participants had difficulty in understanding why fining an organization and thus increasing its financial instability was the right way to promote better IPC practice. An especially important consequence of a highly punitive approach to performance management was the creation of a culture of fearfulness. Participants reported that it contributed to the suppression of a learning culture, made people timid, and inhibited joy in work.

These findings suggest that better recognition is needed of the potential and the limits of performance management. One possibility for preserving some of the advantages of this approach, while reducing its negative effects, may lie in collaborative models that seek to promote infection control, while simultaneously retaining professional support.^32^ Approaches based on professional communities may be especially promising in their ability to address well-known problems of attempts to change practice among professionals, since they are more likely to be favourably disposed to ‘directions for performance’ coming from their peers.^33^ An emphasis on co-operation and norms of reciprocity rather than administrative fiats and managerial instructions may help to enable collaborative activity to be maintained over the longer term and to promote sustainability.^34^ In achieving such co-operation, studies point to the importance of clinical and organizational leadership, a collective focus on patient safety, and an affirming emotional context.^35^ For instance, the iconic Michigan Keystone programme appeared to achieve very substantial and impressive reductions in central line infections in ICUs without resorting to punitive measures.^20^

It has remained difficult to assess the potential of such campaign or community-style approaches in England. First, many of the initiatives, whereas they appeared to be voluntary in principle, were run on government platforms and thus found it difficult to escape the performance management taint.^18^ Second, some of the more genuinely voluntary initiatives remained unevaluated. Third, it was virtually impossible to detect the effects of any specific initiative because each was taking place in a context of multiple other policy pressures for improvement.^36^ Further programmes and studies should address these deficits. Though performance management has clearly had an important role in the history of IPC in England and will continue to have some role in the future, persisting with harsh regimes risks souring relationships and increasing resistance to continued improvement efforts. Nonetheless, there is a real risk that local teams may be left to flounder in the absence of external imperatives that can bring pressure to bear on organizational leaders.

## Figures and Tables

**Table I:** Selected policy events relevant to HCAIs in the UK, 2001–2011

Year	Policy event
2001	Mandatory surveillance of MRSA bacteraemia
2001	Publication of first edition of ‘epic’, the national guidelines for preventing HCAIs
2003	Report of the Chief Medical Officer, *Winning ways*: guidance to reduce HCAIs in England
2004	Mandatory surveillance of *Clostridium difficile* infections in patients aged >65 years, extended to all ages in 2007
2004	Mandatory surveillance of orthopaedic surgical site infections
2004	All NHS organizations required to appoint a director of IPC
2004	Launch of *cleanyourhands* campaign in England and Wales funded by Department of Health and co-ordinated by the National Patient Safety Agency
2004	Patient Safety Alert issued, mandating placement of bedside alcohol hand rub
2005	*Saving lives*: a delivery programme launched to reduce HCAI, including MRSA campaign, involving high-impact interventions based on the care bundle principle
2006	Health Act 2006: code of practice for the prevention and control of HCAIs, introducing requirement for provider registration with regulator, requirement for providers to ensure protection against HCAI, and new code of practice on infections
2006	Visits by Department of Health improvement teams to acute hospitals
2006	Chief Medical Officer makes Chief Executive Officers personally responsible for the accuracy of infection data submitted by their organizations
2007	Introduction of ‘bare below the elbows’ guidance
2008	Health and Social Care Act 2008. Required registration with the Care Quality Commission: duty to protect patients against HCAIs. New code of practice.
2008	Prime Minister declares HCAIs a ‘top priority’ and orders a programme of deep cleaning
2008	Patient Safety First campaign
2008	National target to reduce *C. difficile* infection by at least 30% by 2011
2009	Some NHS organizations participated in CQUIN (Commissioning for Quality and Innovation) schemes that made a percentage of their incomes dependent on demonstrating compliance
2009	Launch of Matching Michigan programme
2011	Mandatory reporting of *Escherichia coli* and meticillin-susceptible *Staphylococcus aureus*

HCAI, healthcare-associated infection; MRSA, meticillin-resistant *Staphylococcus aureus*; NHS, National Health Service; IPC, infection prevention and control.
